# CD27^+^CD38^hi^ B Cell Frequency During Remission Predicts Relapsing Disease in Granulomatosis With Polyangiitis Patients

**DOI:** 10.3389/fimmu.2019.02221

**Published:** 2019-09-24

**Authors:** Anouk von Borstel, Judith Land, Wayel H. Abdulahad, Abraham Rutgers, Coen A. Stegeman, Arjan Diepstra, Peter Heeringa, Jan Stephan Sanders

**Affiliations:** ^1^Division of Nephrology, Department of Internal Medicine, University Medical Center Groningen, University of Groningen, Groningen, Netherlands; ^2^Department of Rheumatology and Clinical Immunology, University Medical Center Groningen, University of Groningen, Groningen, Netherlands; ^3^Department of Pathology and Medical Biology, University Medical Center Groningen, University of Groningen, Groningen, Netherlands

**Keywords:** vasculitis, granulomatosis with polyangiitis, ANCA, B cells, relapse

## Abstract

**Background:** Granulomatosis with polyangiitis (GPA) patients are prone to disease relapses. We aimed to determine whether GPA patients at risk for relapse can be identified by differences in B cell subset frequencies.

**Methods:** Eighty-five GPA patients were monitored for a median period of 3.1 years (range: 0.1–6.3). Circulating B cell subset frequencies were analyzed by flow cytometry determining the expression of CD19, CD38, and CD27. B cell subset frequencies at the time of inclusion of future-relapsing (F-R) and non-relapsing (N-R) patients were compared and related to relapse-free survival. Additionally, CD27^+^CD38^hi^ B cells were assessed in urine and kidney biopsies from active anti-neutrophil cytoplasmic autoantibody-associated vasculitides (AAV) patients with renal involvement.

**Results:** Within 1.6 years, 30% of patients experienced a relapse. The CD27^+^CD38^hi^ B cell frequency at the time of inclusion was increased in F-R (median: 2.39%) compared to N-R patients (median: 1.03%; *p* = 0.0025) and a trend was found compared with the HCs (median: 1.33%; *p* = 0.08). This increased CD27^+^CD38^hi^ B cell frequency at inclusion was correlated to decreased relapse-free survival in GPA patients. In addition, 74.7% of patients with an increased CD27^+^CD38^hi^ B cell frequency (≥2.39%) relapsed during follow-up compared to 19.7% of patients with a CD27^+^CD38^hi^ B cell frequency of <2.39%. No correlations were found between CD27^+^CD38^hi^ B cells and ANCA levels. CD27^+^CD38^hi^ B cell frequencies were increased in urine compared to the circulation, and were also detected in kidney biopsies, which may indicate CD27^+^CD38^hi^ B cell migration during active disease.

**Conclusions:** Our data suggests that having an increased frequency of circulating CD27^+^CD38^hi^ B cells during remission is related to a higher relapse risk in GPA patients, and therefore might be a potential marker to identify those GPA patients at risk for relapse.

## Introduction

Anti-neutrophil cytoplasmic autoantibody (ANCA)-associated vasculitides (AAV) are autoimmune diseases involving small- to medium-sized blood vessels (1). AAV are classified into granulomatosis with polyangiitis (GPA), microscopic polyangiitis (MPA), and eosinophilic GPA (EGPA) (2). In these patients, ANCA directed against proteinase 3 (PR3) (3) or myeloperoxidase (MPO) (4) are frequently present. PR3-ANCA are detected in the majority of GPA patients, whereas MPO-ANCA are more often detected in MPA and EGPA patients. PR3-ANCA-positive patients have a significantly increased relapse rate compared to MPO-ANCA-positive patients. Approximately 50% of the PR3-ANCA-positive patients experience a relapse within 4 years of follow-up, compared to 25% of the MPO-ANCA-positive patients (5). Predicting relapses remains pivotal for patient care, as relapses are associated with considerable morbidity caused by both disease- and therapy-related damage. So far, no biomarker for the prediction of relapse has been found to be reliable in GPA patients. Combined data of multiple studies has shown that ANCA titers can predict future relapses only to a limited extent (6).

The clinical benefit observed in B cell depletion trials using rituximab in patients with AAV strongly supports the contention that B cells are key contributors in the AAV immunopathogenesis (7–9). ANCA were detectable in the circulation of AAV patients in remission and decreased 6 months after start of rituximab treatment (7, 8). This suggests that clinical improvement seen in rituximab-treated AAV patients precedes the reduction of ANCA titers. Although disease relapses did occur in rituximab-treated patients, these occurred only after B cell repopulation (8). Together these results indicate that B cells may contribute to the disease pathogenesis and relapses in an antibody-independent manner. Importantly, B cells are the precursors of antibody- and ANCA-producing plasma cells and might be correlated to the occurrence of disease relapses in GPA patients. However, the exact B cell subtype that can be used to predict GPA relapses is undefined.

The B cell subset distribution in GPA patients has been found to differ from that in healthy controls (HCs). Compared to HCs, the frequencies of CD27^+^CD38^−/+^ memory and CD24^hi^CD27^+^ B cells were decreased, whereas CD27^−^CD38^−/+^ naïve B cells were increased in GPA patients (10). In addition, the circulating B cell phenotype of active GPA patients showed decreased frequencies of CD24^hi^CD38^hi^ and CD27^−^CD38^hi^ transitional B cells compared to remission patients (10). To our knowledge, no studies assessed the CD27^+^CD38^hi^ B cell frequencies in GPA patients. Monitoring the B cell distribution in peripheral blood might be a good prognostic tool to predict disease relapses and could perhaps be more efficient than measuring serum ANCA levels.

This study aimed to elucidate whether a specific subset of B cells could be identified that is associated with risk for relapse in GPA patients, and if monitoring these B cells could predict the disease course in these patients. Furthermore, we assessed B cell migration to the kidneys of active GPA patients with renal involvement. B cell subset frequencies were determined in GPA patients in remission, with and without future disease relapses, and their relation to disease relapses and serum ANCA levels was analyzed. Additionally, the B cell phenotype was investigated in matched urine and blood samples and kidney biopsies from active AAV patients with renal involvement.

## Methods

### Study Population

Eighty-five GPA patients and 48 age-matched HCs (52.1% male, median age: 57.3 years, range: 40–74) were enrolled in the study between 2010 and 2013. This GPA patient cohort was prospectively monitored. GPA diagnosis was based on definitions described in the Chapel Hill Consensus Conference (2). At inclusion, all patients were in complete remission [Birmingham vasculitis activity score (BVAS) = 0]. Patients tested PR3-ANCA-positive at least once during their disease course. None of the patients or controls experienced an infection at the time of sampling. Only patients that were at least ten months post-rituximab treatment were included. Patients that experienced a relapse during follow-up were designated to the future-relapsing (F-R) group and the patients remaining in remission were assigned to the non-relapsing (N-R) group. Diagnosis of disease relapse was based on clinical judgment and had to result in initiation/increase of immunosuppressive treatment. Relapses were monitored in all patients and median time between sampling and diagnosis of relapse was 1.6 years (range: 0.1–3.8), whereas the total follow-up time for N-R patients was 3.3 years (range: 1.3–6.3). The main clinical and laboratory data of the patients are summarized in Table 1. All patients and HCs provided informed consent and all experimental procedures were conducted according to the policies of the UMCG. The medical ethics committee of the UMCG (Groningen, the Netherlands) approved the study.

**Table 1:** Clinical data and characteristics of GPA patients.

	**GPA-no relapse during follow-up** **(N-R)**	**GPA-relapsed during follow-up** **(F-R)**	***P*-value (N-R vs. F-R)**
Subjects, *n* (% male)	58 (39.7)	27 (44.4)	0.7799
Age, mean (range)	59 (26–84)	55 (30–81)	0.3157
cANCA titer, median (range)	1:40 (0–1:640)	1:80 (0–1:640)	0.3149
cANCA positive (>1:20), *n* (%)	42 (66.7)	20 (74.1)	0.3478
Creatinine μmol/L, median (range)	72 (20–147)	73 (21–171)	0.2167
CRP mg/L, median (range)	4.9 (0.5–20)	4.9 (0.4–83)	0.5286
Disease duration in years, median (range)	9.3 (1.4–42.1)	11.4 (2.1–28.7)	0.3015
Number of total relapses before inclusion, median (range)	1 (0–6)	3 (0–10)	**0.0001**
Lymphocyte count * 10^6^/L, median (range)	1,200 (340–2900)	695 (240–1,640)	**0.003**
B cell count * 10^6^/L, median (range)	91 (4.1–510.8)	33.7 (1.3–246)	**0.0017**
CD19^+^ B cells (%), median (range)	8.1 (0.7–22.2)	3.9 (0.13–21.1)	0.0785
IS therapy at time of sampling, *n* (%)	22 (37.9)	19 (70.4)	**0.0053**
Azathioprine, *n* (%)	4 (6.8)	8 (29.6)	**0.0051**
Azathioprine + prednisolone, *n* (%)	8 (13.8)	6 (22.2)	0.3293
Cyclophosphamide + prednisolone, *n* (%)	1 (1.7)	0 (0)	0.4925
Mycophenolate mofetil + prednisolone, *n* (%)	3 (5.2)	4 (14.8)	0.1322
Prednisolone, *n* (%)	6 (10.3)	1 (3.7)	0.2998
Induction therapy			
Azathioprine + prednisone, *n* (%)	2 (3.5)	0 (0)	0.3288
Cyclophosphamide + prednisone, *n* (%)	50 (86.2)	26 (96.3)	0.1593
Methotrexate + prednisone, *n* (%)	2 (3.5)	0 (0)	0.3288
Mycophenolate mofetil + prednisone, *n* (%)	0 (0)	1 (3.7)	0.1404
Cotrimoxazole, *n* (%)	4 (6.8)	0 (0)	0.1622
No. clinical manifestations baseline, median (range)	3 (1–6)	4 (1–6)	**0.0104**
Kidney involvement, *n* (%)	31 (57.1)	19 (70.4)	0.14
Airway involvement, *n* (%)	53 (91.4)	26 (96.3)	0.41

*AAV, ANCA-associated vasculitides; cANCA, cytoplasmic anti-neutrophil cytoplasmic autoantibody; CRP, C-reactive protein; F-R, future-relapsing; IS, immunosuppressive; N-R, non-relapsing; No., number. The bold values are the p-values that indicate a significant difference between the F-R and N-R group*.

In addition to patients in remission, eleven AAV patients (seven GPA and three MPA; Table 2) with clinical signs and symptoms of active vasculitis with renal involvement were included to assess their urine and blood samples simultaneously. After additional diagnostic investigation, renal disease could be confirmed for seven AAV patients (five GPA and two MPA). Four active GPA patients with renal involvement were included to assess plasma cell infiltration in kidney biopsies (Table 2).

**Table 2:** Clinical data and characteristics of AAV patients with active disease and signs of renal involvement.

	**Urine analysis**	**Plasma cell histology**
Disease subtype, *n* (% male)	MPA, 2 (50)/GPA, 5 (100)	GPA, 4 (100)
Age, median (range)	68 (59.9–82)	71.1 (59–86.4)
ANCA positive, *n* (%)	7 (100)	4 (100)
BVAS, median (range)	12 (11–21)	13 (11–15)
Creatinine umol/L, median (range)	174 (94–483)	236.5 (165–566)
CRP mg/L, median (range)	41 (6–85)	22 (6–85)
Proteinuria urine g/L, median (range)	1.22 (0.4–3.57)	2.5 (0.87–3.57^*^)
IS therapy, *n* (%)	3 (42.9)	2 (50)
No. clinical manifestations, median (range)	2 (1–4)	2 (1–2)

BVAS, Birmingham Vasculitis Activity Score; cANCA, cytoplasmic anti-neutrophil cytoplasmic autoantibody; CRP, c-reactive protein; GPA, granulomatosis with polyangiitis; IS, immunosuppressive; MPA, microscopic polyangiitis; No., number;

* *not determined for 1 patient*.

### Flow Cytometry Analysis of B Cell Subsets

**Figure 1:**
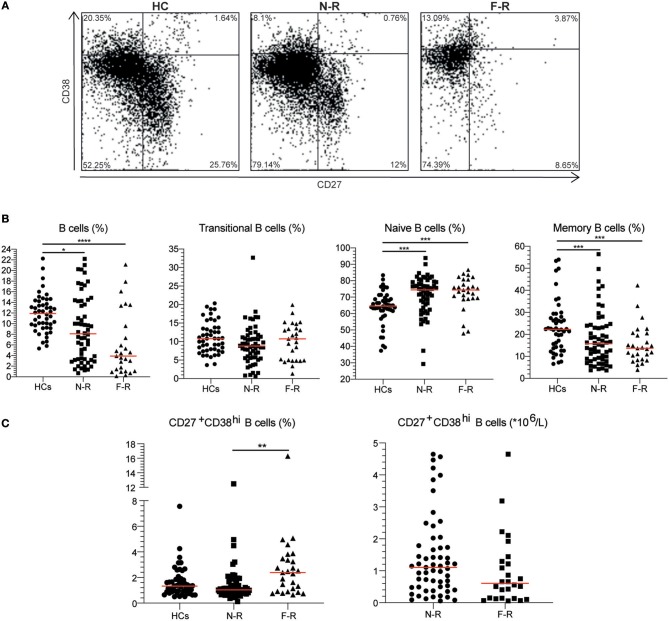
The CD27^+^CD38^hi^ B cell frequency is increased in F-R patients. **(A)** Based on CD27 and CD38 expression, four subsets were distinguished: naive B cells as CD27^−^CD38^−/dim^, transitional B cells as CD27^−^CD38^hi^, memory B cells as CD27^+^CD38^−/dim^ and CD27^+^CD38^hi^ B cells in HCs, N-R patients and F-R patients. **(B)** The proportions of total B cells, transitional B cells, naïve B cells, and memory B cells are depicted for HCs, N-R patients and F-R patients. **(C)** The CD27^+^CD38^hi^ B cell frequency is expressed as a percentage within total B cells. The absolute CD27^+^CD38^hi^ B cell count was calculated using the lymphocyte count and CD27^+^CD38^hi^ B cell frequency in F-R and N-R patients and is given as count * 10^6^/L peripheral blood. Red bars represent the median value. ^*^p < 0.05; ^**^p < 0.01; ^***^p < 0.001; ^****^p < 0.0001.

EDTA venous blood was obtained from GPA patients and HCs. Immediately after sampling, blood was washed twice in PBS with 1% BSA (wash buffer). Next, 100 μl cell suspension was stained using anti-human CD19-eFluor450, CD27-APC-eFluor780, CD38-PE-Cy7 (eBiocience, San Diego, CA, USA) or the corresponding isotype controls. Cells were treated with 10x FACS Lysing solution (BD Biosciences, San Jose, CA, USA) and acquired on an LSR-II flow cytometer (BD Biosciences). For all flow cytometry analyses, data were collected for at least 2^*^10^5^ cells, and analyzed using Kaluza 1.5a software (Beckman Coulter, Brea, CA, USA). Supplementary Figure 1 shows the gating strategy and Figure 1A the representative gating examples for each group. The B cell subset percentages were converted to absolute numbers using the lymphocyte count and the percentage of total B cells. No lymphocyte counts were available for the HC group.

### Flow Cytometry Analysis of CD27^+^CD38^hi^ B Cells in Blood and Urine

Urine and blood samples were collected from ten AAV patients with active disease. Urine samples were prepared as described previously (11). Briefly, urine was diluted 1:1 in PBS and centrifuged at 1,800 rpm. The sediment was resuspended in PBS and mononuclear cells (MNCs) were isolated using lymphoprep (Axis-Shield, Oslo, Norway). Next, MNCs were resuspended in wash buffer and stained with anti-human CD19-PerCP-Cy5.5, CD45-BV605, CD27-APC (BioLegend, San Diego, CA, USA), CD3-BUV395, and CD38-BB515 (BD Biosciences) for 15 min at room temperature in the dark. Isotype-matched non-specific antibodies were used as negative controls. In parallel, blood samples were labeled with the aforementioned monoclonal antibodies. Afterwards, cells were treated with 10x diluted FACS lysing solution for 10 min, washed twice in wash buffer and immediately analyzed. Stained urine and blood samples were acquired on the LSR-II and data was analyzed using Kaluza 1.5a software. **Figure 3A** shows a representative gating example of both blood and urine. Three patients were excluded because no renal involvement was diagnosed and accordingly no B cells were present in the urine.

### Analysis of Plasma Cells in Kidney Biopsies

CD27^+^CD38^hi^ B cells likely represent plasmablasts and/or plasma cells (12, 13), however, determining CD38^hi^ expressing B cells in tissue is impossible as CD38 expression is not unique for plasmablasts and distinguishing CD38^+^ and CD38^hi^ expression visually is arbitrary. Thus, kidney biopsies of four active GPA patients with renal involvement were stained for plasmablast and plasma cell infiltration using a MUM1/IRF4 monoclonal antibody (clone MUM1p, DAKO, Santa Clara, CA, USA) in an automated stainer (Ventana, Roche, Basel, Switzerland).

### Serum ANCA Levels

ANCA detection was performed by indirect immunofluorescence, as described previously (14). ANCA titers of 1:40 or higher were considered positive. Serum PR3-ANCA levels could be determined in samples of 51 patients by Phadia ImmunoCAP® 250 analyzer using EliA PR3^S^ (Thermo Fisher Scientific, Waltham, MA, USA).

### *In vitro* PR3-ANCA and IgG

Peripheral blood mononuclear cells (PBMCs) were isolated and cultured as described before (15). In short, for 79 GPA patients PBMCs were available and were cultured for 12 days with or without 3.2 μg/mL CpG-ODN 2006 (Hycult Biotech, Uden, the Netherlands), 100 ng/mL IL-21 (Immunotools, Friesoythe, Germany) and 100 ng/mL BAFF (PeproTech Inc., Rocky Hill, CT, USA). Stimulated and spontaneous PR3-ANCA (RU/mL) production was determined in the supernatant by Phadia ImmunoCAP® 250 analyzer using EliA PR3^S^ (Thermo Fisher Scientific) and total spontaneous and stimulated IgG production was assessed by ELISA. Five samples (2 F-R and 3 N-R) were excluded because of a culture infection.

### Statistical Analysis

Statistical analysis was performed using GraphPad Prism v7.0 (GraphPad Software, San Diego, CA, USA) and SPSS (IBM Corporation, Chicago, IL, USA). B cell subset frequencies of HCs, F-R and N-R GPA patients were compared using a Kruskal Wallis test. Individual groups were compared with Dunn's multiple comparison test. The plasmablast counts were compared using the Mann Whitney *U*-test. Furthermore, a Kaplan Meier curve of probability of relapse-free survival was plotted for patients with less or more than 2.39% CD27^+^CD38^hi^ B cells (one deceased patient was excluded) and CD27^+^CD38^hi^ B cell frequency was compared between inclusion samples and samples before relapse of F-R patients using the Wilcoxon signed rank test. For F-R patients the mean difference between inclusion and the last sample taken before relapse was 0.8 ± 0.5 years. Cox regression analysis was performed to determine the relation between log-transformed CD27^+^CD38^hi^ B cells and immunosuppressive treatment and relapse occurrence.

Correlations between different parameters were determined with the Spearman r correlation. Finally, relapse-free survival was determined using the Log rank test. *P* <0.05 were considered statistically significant.

## Results

### Clinical Patient Characteristics

During a median follow-up of 1.6 years, 27 GPA patients experienced a disease relapse (future-relapsing; F-R), whereas 58 patients did not (non-relapsing; N-R). The number of clinical manifestations at first diagnosis was higher in F-R compared to N-R patients (Table 1). Additionally, F-R patients had experienced more relapses than N-R patients before study inclusion. At inclusion, more F-R patients received immunosuppressive therapy. The cANCA titers and frequency of patients with a positive cANCA titer did not differ between F-R and N-R patients.

Induction therapy and other baseline clinical characteristics such as C-reactive protein levels and serum creatinine did not differ significantly between both patient groups (Table 1).

### Increased Frequencies of Circulating CD27^+^CD38^hi^ B Cells in F-R Patients

The B cell phenotype was determined in 27 F-R and 58 N-R patients in remission and in 48 HCs. For the F-R patient group a median of 1,463 B cell events (range: 49–7,913) and for the N-R patient group a median of 3,030 B cell events (range: 255–8,321) were acquired on the flow cytometer. The total B cell frequency was significantly lower in both patient groups compared to HCs (Table 1 and Figure 1B). Analyzing B cell subsets, the transitional B cell frequencies were not different between the groups. The naïve B cell frequency was higher in both N-R and F-R patients compared to HCs and the percentage of memory B cells was lower in both patients groups as compared to HCs (Figure 1B), which is in line with previously published results (10). The circulating CD27^+^CD38^hi^ B cell percentage was significantly increased in F-R patients compared to N-R patients, while N-R patients were similar to HCs (Figure 1C).

The lymphocyte and B cell count were significantly decreased in F-R patients (Table 1). Using the lymphocyte count we calculated the absolute numbers of circulating CD27^+^CD38^hi^ B cells. No differences in numbers of circulating CD27^+^CD38^hi^ B cells were observed between both patient groups (Figure 1C).

Cox regression analysis demonstrated a significant relation between (log transformed) CD27^+^CD38^hi^ B cells and relapse, whereas only a trend between the use of immunosuppressive medication and relapse was observed (Supplementary Table 1).

### Increased Circulating CD27^+^CD38^hi^ B Cell Frequency in GPA Patients Is Related to Decreased Relapse-Free Survival

**Figure 2:**
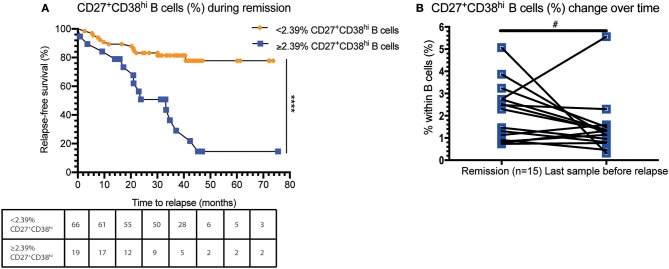
An increased CD27^+^CD38^hi^ B cell frequency is related to decreased relapse-free survival. **(A)** Percentage relapse-free survival is depicted in patients in remission with < or ≥2.39% circulating CD27^+^CD38^hi^ B cells (i.e., median value of F-R patients). Hazard ratio of 8.8 (95% CI: 3.35–23.2). The number of subjects at risk are given for each time point in the table. **(B)** The CD27^+^CD38^hi^ B cell frequency is expressed as a percentage within B cells during remission and 1–3 months before relapse (*n* = 15). ^#^*p* = 0.10, ^****^p < 0.0001.

Subsequently, we investigated whether an increased CD27^+^CD38^hi^ B cell frequency was related to future disease relapse. We divided the GPA patients into two groups based on the median CD27^+^CD38^hi^ B cell frequency of the F-R patients: one group consisted of patients with <2.39% and the other of patients with 2.39% or more CD27^+^CD38^hi^ B cells. Significantly more patients with a high percentage of CD27^+^CD38^hi^ B cells experienced a future disease relapse than patients with a low CD27^+^CD38^hi^ B cell frequency (Figure 2A). The percentage of patients (80.3%) who remained relapse-free during follow-up was significantly higher in GPA patients with <2.39% circulating CD27^+^CD38^hi^ B cells compared to patients with ≥2.39% CD27^+^CD38^hi^ B cells (25.3%).

Moreover, during follow-up the circulating CD27^+^CD38^hi^ B-cell frequency in F-R patients tended to decrease 1–3 months prior to relapse compared to remission samples (Figure 2B).

### CD27^+^CD38^hi^ B Cells Are Present in the Kidney and Increased in the Urine of Active AAV Patients With Renal Involvement

**Figure 3:**
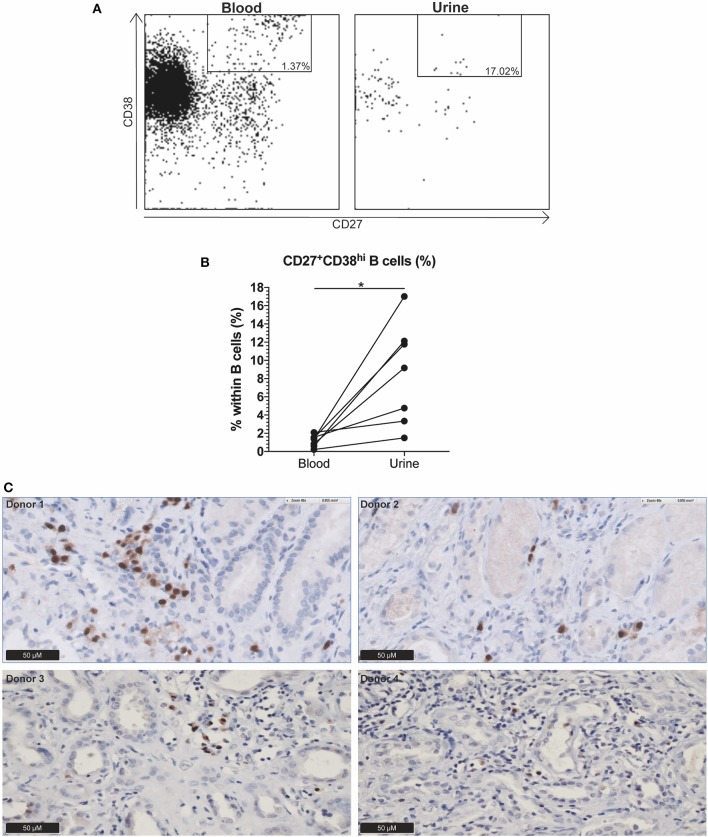
Indication that CD27^+^CD38^hi^ B cells migrate from the circulation to the kidney in active AAV patients with renal involvement. **(A)** Using CD27 and CD38, the CD27^+^CD38^hi^ B cell subset could be distinguished in both urine (right) and peripheral blood (left). **(B)** Circulating and urine CD27^+^CD38^hi^ B cell percentage in active patients with renal involvement (*n* = 4). **(C)** Immunohistochemistry for MUM1/IRF4, showing presence of plasma cells in formalin fixed paraffin embedded renal biopsy tissue samples of four patients. ^*^p = 0.03.

As the circulating CD27^+^CD38^hi^ B cell frequency seemed to decrease in GPA patients before relapse whereas this did not occur in N-R patients over a period of 12 months (data not shown), we hypothesized that CD27^+^CD38^hi^ B cells migrate to sites of inflammation. To explore this, we analyzed the CD27^+^CD38^hi^ B cell frequency in both the circulation and urine of 10 active AAV patients. None of the three patients without active renal involvement presented with B cells in the urine. Whilst in the seven patients with active renal involvement B cells could be detected in the urine, and contained an increased proportion of CD27^+^CD38^hi^ B cells compared to the circulation (Figure 3B). Since CD27^+^CD38^hi^ B cells likely represent plasmablasts and plasma cells (12, 13) and proper evaluation of CD38^hi^ expressing B cells is impossible in tissue, we stained four kidney biopsies using an antibody that detects the MUM1/IRF4 transcription factor that is specifically expressed in plasmablasts and plasma cells. Figure 3C demonstrates plasma cell infiltration in the kidneys of four active GPA patients. For two of these patients, the urinary CD27^+^CD38^hi^ B cell frequency was determined and this frequency was increased compared to the circulation (Figure 3B). These findings might indicate that CD27^+^CD38^hi^ B cells migrate from the circulation to inflamed kidneys during active disease.

### CD27^+^CD38^hi^ B Cell Frequency Is Not Related to ANCA Levels in GPA Patients

As CD27^+^CD38^hi^ B cells are likely the precursors of antibody-producing plasma cells, and since we found evidence that the CD27^+^CD38^hi^ B cell frequency is related to future relapses, we examined whether CD27^+^CD38^hi^ B cell numbers and percentages correlated with ANCA and total IgG levels. Such a correlation could indicate that the increased CD27^+^CD38^hi^ B cell percentages are disease specific. No significant correlation was found between the percentage of circulating CD27^+^CD38^hi^ B cells and ANCA titers (Supplementary Figure 2A). Furthermore, no positive correlation was found between serum PR3-ANCA levels, and the percentage or number (data not shown) of circulating CD27^+^CD38^hi^ B cells (Supplementary Figure 2B). Similarly, the CD27^+^CD38^hi^ B cell frequency did not correlate with either spontaneous *in vitro* produced (Supplementary Figure 2C), stimulated *in vitro* produced PR3-ANCA (Supplementary Figure 2D), or total *in vitro* spontaneous or stimulated IgG (data not shown).

## Discussion

In this study, we demonstrate an increase in the frequency of circulating CD27^+^CD38^hi^ B cells in GPA patients with future relapses, and their association with an increased relapse risk. We found that the circulating CD27^+^CD38^hi^ B cell frequency tended to decrease with impending relapse and detected CD27^+^CD38^hi^ B cells in the kidneys and urine of active patients with renal involvement, suggesting migration of plasmablasts to inflamed organs.

So far, multiple predictors of (future) disease activity have been proposed, including platelet count (16) and lung involvement at diagnosis (17). However, those markers are not ideal since they only identify active disease or focus on a subpopulation of patients. Currently, ANCA levels are the best biomarker available to identify approaching disease relapses (6). However, a disadvantage of ANCA levels as a biomarker is that it is not reliable in all patients, which highlights the need for better indicators of future disease activity. Here, we found that GPA patients with increased frequencies of circulating CD27^+^CD38^hi^ B cells during remission were more likely to relapse in the (near) future.

As circulating CD27^+^CD38^hi^ B cells are likely the direct precursors of (auto)antibody-producing plasma cells (18, 19), they may play a major role in GPA pathogenesis. In other autoimmune diseases such as SLE (20, 21), IgG4-related disease (22), and anti-PLA2R1 related membranous nephropathy (23) the plasmablast frequency has been reported to be related to disease activity, as were absolute plasmablast numbers in SLE (20) and IgG4-related disease (22). Additionally, plasmablast frequencies and numbers correlated significantly with (auto)antibody levels in IgG4-related disease (22) and SLE (20), although in one study on IgG4-related disease such a correlation could not be confirmed (24). Interestingly, in RA patients sorted plasmablasts were found to produce anti-citrullinated protein antibodies (ACPA) with and without *in vitro* stimulation. However, the authors did not study whether the circulating plasmablasts correlated to serum ACPA levels (25). In the current study we did not find a correlation between CD27^+^CD38^hi^ B cells and ANCA titers, serum PR3-ANCA levels or spontaneous and stimulated *in vitro* produced PR3-ANCA. Previously it was shown that PR3-specific B cells could be identified among switched memory B cells and plasmablasts in higher numbers in active and remission PR3-ANCA patients compared to HCs (26). In the current study however, we compared secreted PR3-ANCA levels, whereas in the study by Cornec et al. (26) B cells binding to PR3 were stained. It might be that not all CD27^+^CD38^hi^ B cells have differentiated into plasma cells and/or must migrate to the bone marrow in order to produce ANCA. Of note, although CD27^+^CD38^hi^ expression is found on precursors of plasma cells (12, 13), these markers may also be expressed on memory B cells (27). Another explanation might be that an increase in CD27^+^CD38^hi^ B cells is not disease-specific but a consequence of smoldering inflammation. Indeed, plasmablasts of IgG4-related disease patients express increased RNA levels of CD44 and SDC1 associated with cell migration and increased surface marker levels of activation markers such as HLA-DR, CD95. and CD86 (22). Additional evidence suggests that elevated IL-6 production by plasmablasts from RA patients induced increased differentiation of T follicular helper cells, and that treatment of RA patients with Tocilizumab decreased the circulating T follicular helper cells in these patients (28). Previously, we have also found that *in vitro* PR3-ANCA production is not related to approaching relapses (29), which may also explain the lack of correlation between CD27^+^CD38^hi^ B cells and *in vitro* ANCA production.

We demonstrated increased CD27^+^CD38^hi^ B cell frequencies in the kidney and urine of renal active AAV patients compared to the circulation, which might indicate CD27^+^CD38^hi^ B cell migration from the circulation to the inflamed kidney. Flow cytometric analysis demonstrated that the B cell subset distribution in the circulation is different from that in the urine samples, which suggests that there is active migration to the kidney instead of leakage of CD27^+^CD38^hi^ B cells into the urine. In line with these observations are data from murine lupus mouse models and SLE patients showing the presence of plasma cells in the inflamed kidney (30, 31) and CXCR4^+^ plasma cells and plasmablasts in renal biopsies, respectively (32). In Kawasaki disease, IgA plasma cell infiltration has been demonstrated in the inflamed kidney as well as in other affected organs (e.g., arteries and lungs) (33). Although B cell and plasma cell infiltration in lesions of GPA patients has been demonstrated before (34), to our knowledge, we are the first to describe plasma cell infiltration into the inflamed kidney in GPA. A recent article by Brix et al. described B cell infiltrates in kidney biopsies from active ANCA-associated glomerulonephritis patients and also found an association of ectopic lymphoid structures with end stage renal disease during follow-up (35). Identification of CD27^+^CD38^hi^ B cells by immunohistochemistry is technically challenging, since double staining of the cell membrane is difficult to evaluate. In addition, it is not possible to make a clear distinction between positive and high CD38 expression. We used the MUM1/IRF4 protein as a surrogate marker since it has the highest expression in plasmablasts and plasma cells (36) and does not stain other cells in kidney tissue.

An important limitation of the current study is the assessment of total CD27^+^CD38^hi^ B cells rather than PR3-specific B cells. Additional limitations include the limited number of patients in the study, the study design as it was performed in a single center, and the limited material that could be obtained for each patient resulting in a low number of B cells that could be acquired in the flow cytometry experiments. Importantly, a confounding factor might be recent vaccinations as this might influence circulating CD27^+^CD38^hi^ B cell levels and this was not tracked by the hospital. Future research should aim to include more patients to assess the extent of plasma cell infiltration in the kidney and the frequency of urinary CD27^+^CD38^hi^ B cells and track recent vaccinations.

In conclusion, in GPA patients an increased CD27^+^CD38^hi^ B cell frequency during remission might be associated with future relapses of disease. Additional prospective studies in larger GPA patient cohorts are needed to fully establish whether the circulating CD27^+^CD38^hi^ B cell frequency during remission constitutes a novel prognostic marker of disease activity.

## Data Availability

The datasets generated for this study are available on request to the corresponding author.

## Ethics Statement

This study was carried out in accordance with the recommendations of the Medical Ethics Committee of the University Medical Center Groningen, with written informed consent from all subjects. All subjects gave written informed consent in accordance with the Declaration of Helsinki. The protocol was approved by the Medical Ethics Committee of the University Medical Center Groningen.

## Author Contributions

AB, AD, and JL carried out experiments, analyzed the data, and made the figures. All authors designed the study, drafted and revised the paper, and approved the final version of the manuscript.

### Conflict of Interest Statement

The authors declare that the research was conducted in the absence of any commercial or financial relationships that could be construed as a potential conflict of interest.
