# Supplementary material for: CD27+CD38hi B Cell Frequency During Remission Predicts Relapsing Disease in Granulomatosis With Polyangiitis Patients
Source: Front Immunol. 2019 Sep 24;10:2221. doi: 10.3389/fimmu.2019.02221 (PMC6769172; doi:10.3389/fimmu.2019.02221)
Supplement: Supplementary file 1 [file Table_1.docx]

**SUPPLEMENTAL MATERIAL**

**Supplementary table 1.** SPSS output of the Cox-regression analysis assessing the relation between both log-transformed CD27^+^CD38^hi^ B cell frequency and immunosuppressive treatment with relapses.

|  | **B** | **SE** | **Wald** | **df** | **Sig.** | **Exp(B)** |
| --- | --- | --- | --- | --- | --- | --- |
| Log-transformed CD27^+^CD38^hi^ B cells (%) | 1.160 | 0.542 | 4.584 | 1 | 0.032 | 3.189 |
| Immunosuppressive treatment | 0.818 | 0.461 | 3.149 | 1 | 0.076 | 2.267 |
