# Supplementary material for: CD27+CD38hi B Cell Frequency During Remission Predicts Relapsing Disease in Granulomatosis With Polyangiitis Patients
Source: Front Immunol. 2019 Sep 24;10:2221. doi: 10.3389/fimmu.2019.02221 (PMC6769172; doi:10.3389/fimmu.2019.02221)
Supplement: Supplementary file 2 [file Data_Sheet_1.docx]

**SUPPLEMENTAL MATERIAL**

CD27

CD19

FSC

CD38

CD19

CD38

CD27

SSC

CD27^+^CD38^hi^ cells

SSC

Memory

Naive

Transitional

**Supplementary figure 1. Gating strategy to determine circulating B-cell subsets.** A gate was first set on the lymphocytes using the FSC/SSC plot. Then, high CD38 expression was determined on total lymphocytes using the CD19/CD38 plot, and CD27^+^ cells were gated on total lymphocytes using the CD19/CD27 plot. Within the lymphocytes we gated on CD19^+^ B-cells, and in the CD27/CD38 plot we determined the different B-cell subsets applying the previously determined gates.
