# Supplementary material for: CD27+CD38hi B Cell Frequency During Remission Predicts Relapsing Disease in Granulomatosis With Polyangiitis Patients
Source: Front Immunol. 2019 Sep 24;10:2221. doi: 10.3389/fimmu.2019.02221 (PMC6769172; doi:10.3389/fimmu.2019.02221)
Supplement: Supplementary file 3 [file Data_Sheet_2.docx]

**SUPPLEMENTAL MATERIAL**


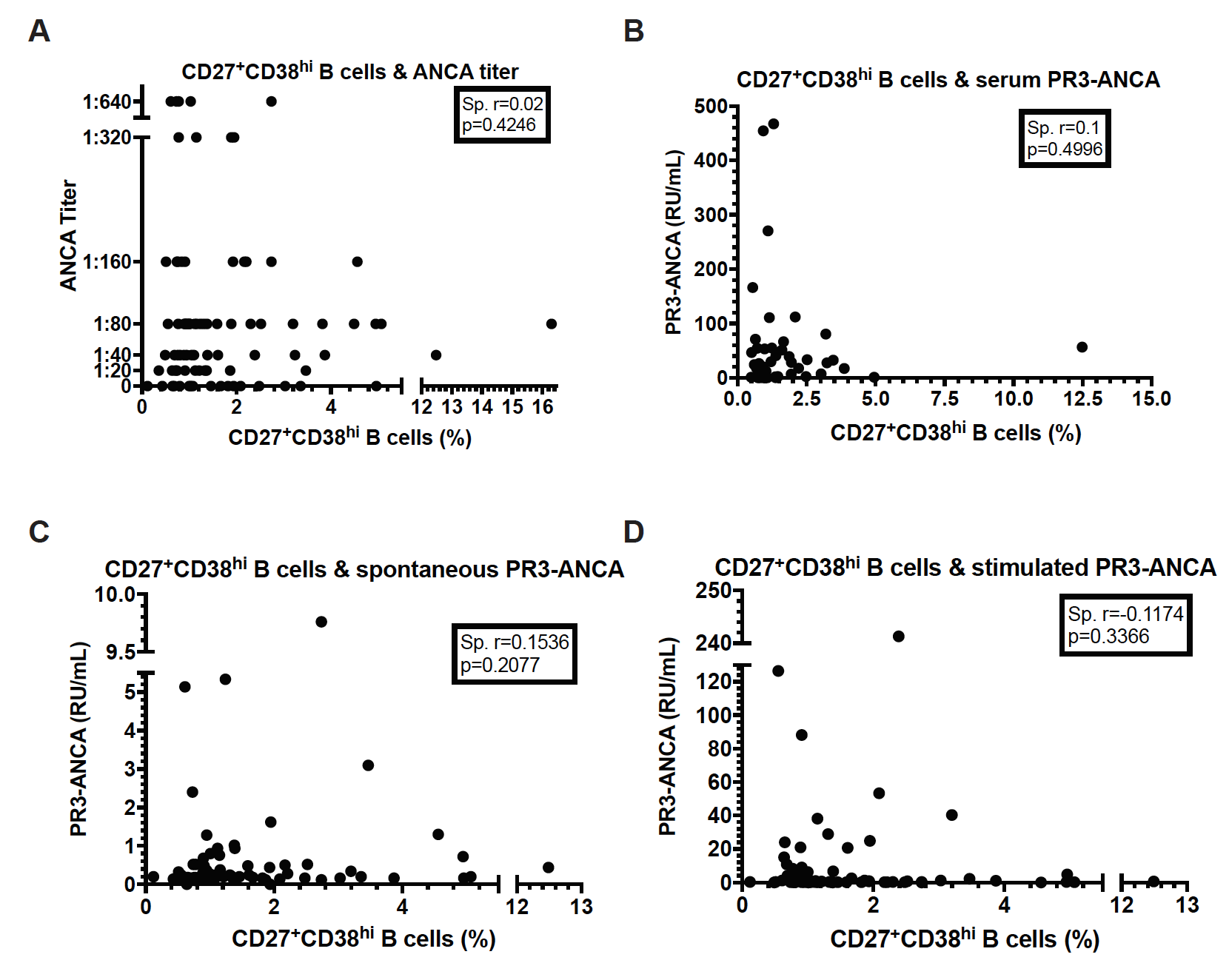


**Supplementary figure 2. The CD27^+^CD38^hi^ B cell frequency does not correlate with ANCA levels.** The CD27^+^CD38^hi^ B cell frequency was correlated to **A.** ANCA titer, **B.** Serum PR3-ANCA levels (IU/mL), **C.** spontaneous *in vitro* produced PR3-ANCA (RU/mL), and **D.** stimulated *in vitro* produced PR3-ANCA (RU/mL). In each figure the Spearman r and p-value are given.
